# Antibody Reactivity to Merozoite Antigens in Ghanaian Adults Correlates With Growth Inhibitory Activity Against *Plasmodium falciparum* in Culture

**DOI:** 10.1093/ofid/ofz254

**Published:** 2019-05-28

**Authors:** Henrietta E Mensah-Brown, Harvey Aspeling-Jones, Rupert K Delimini, Kwaku Poku Asante, Emmanuel Amlabu, Saikou Y Bah, James G Beeson, Gavin J Wright, David J Conway, Gordon A Awandare

**Affiliations:** 1 West African Centre for Cell Biology of Infectious Pathogens, University of Ghana, Legon, Accra, Ghana; 2 Department of Biochemistry, Cell and Molecular Biology, University of Ghana, Legon, Accra, Ghana; 3 London School of Hygiene and Tropical Medicine, London, United Kingdom; 4 Department of Biomedical Sciences, University of Health and Allied Sciences, Ho, Ghana; 5 Kintampo Health Research Center, Kintampo, Ghana; 6 The Burnet Institute for Medical Research and Public Health, Melbourne, Australia; 7 Department of Microbiology, Monash University, Clayton, Australia; 8 Pathogens and Microbes Programme, Wellcome Trust Sanger Institute, United Kingdom; 9 Department of Biochemistry, Kogi State University, Anyigba, Nigeria

**Keywords:** falciparum malaria, growth inhibitory activity, humoral immunity, invasion, vaccine development

## Abstract

**Background:**

*Plasmodium falciparum* uses a repertoire of merozoite-stage proteins for invasion of erythrocytes. Antibodies against some of these proteins halt the replication cycle of the parasite by preventing erythrocyte invasion and are implicated as contributors to protective immunity against malaria.

**Methods:**

We assayed antibody reactivity against a panel of 9 recombinant antigens based on erythrocyte-binding antigen (EBA) and reticulocyte-like homolog (Rh) proteins in plasma from children with malaria and healthy adults residing in 3 endemic areas in Ghana using enzyme-linked immunosorbent assay. Purified immunoglobulin (Ig)G from adult plasma samples was also tested for invasion inhibition against 7 different *P falciparum* culture lines, including clinical isolates.

**Results:**

Antibodies against the antigens increased in an age-dependent manner in children. Breadth of reactivity to the different antigens was strongly associated with in vitro parasite growth inhibitory activity of IgG purified from the adults. The strongest predictors of breadth of antibody reactivity were age and transmission intensity, and a combination of reactivities to Rh2, Rh4, and Rh5 correlated strongly with invasion inhibition.

**Conclusions:**

Growth inhibitory activity was significantly associated with breadth of antibody reactivity to merozoite antigens, encouraging the prospect of a multicomponent blood-stage vaccine.

There are over 200 million cases of malaria annually, resulting in approximately half a million deaths [1], the majority being caused by *Plasmodium falciparum*. The majority of malaria cases and deaths occur in sub-Saharan Africa, affecting mainly children under the age of 5 years and pregnant women [1]. The clinical symptoms of malaria occur as a result of the erythrocytic stage of the parasite’s life cycle, due in part to inflammatory immune responses. The development of a blood-stage vaccine could potentially reduce the incidence of malaria. However, the development of such a vaccine faces many challenges, including the complexity of the immune response and the lack of knowledge of the exact immune mechanism for parasite neutralization. The large number of parasite antigens to which the immune system is exposed, coupled with antigenic variation and polymorphism, makes the robust identification of specific targets of immunity against malaria difficult [2].

Two *P falciparum* protein families, erythrocyte-binding antigens (EBA) and reticulocyte binding-like homolog proteins (Rh), play an essential role in erythrocyte invasion [3, 4]. Because these proteins are important targets of immunity [5–8], they have been evaluated as priority candidate antigens for developing a blood-stage vaccine [9–12]. However, variation in the utilization of different EBA and Rh proteins can mediate immune escape [8], suggesting that vaccines may need to include multiple proteins to maximize efficacy.

Antibodies against EBA and Rh proteins have shown the ability to inhibit parasite growth in vitro [10, 13], supporting their importance as targets of immunity. Passive transfer of immunoglobulin (Ig)G from semi-immune adults to children with malaria can reduce parasitemia and mitigate symptoms [14, 15], so purified IgG from adults may be useful in studying the targets of invasion inhibitory antibodies. In this study, antibodies in children with clinical malaria in 3 areas in Ghana with differing levels of endemicity were assayed against a panel of 9 EBA and Rh recombinant antigens and tested for correlations with age and levels of parasitemia. Separate analyses explored the correlation between ability of purified IgG to inhibit parasite growth in vitro and antibody reactivities to the antigens in semi-immune adults living in a highly endemic area of Ghana.

## METHODS

### Study Sites and Sampling

Children aged between 2 and 14 years with *P falciparum* slide-positive clinical malaria were recruited in 3 ecologically distinct areas in Ghana: (1) Ledzokuku-Krowor municipality (LEKMA) in Accra, (2) Kassena and Nankana districts in northern Ghana, and (3) Kintampo Municipality in the middle belt of Ghana, as described in detail elsewhere [16, 17]. The local malaria transmission intensity as previously measured by the entomological inoculation rates was highest in Kintampo (>250 infective bites/person per year), followed by Navrongo (<250 infective bites/person per year), and lowest in Accra (<50 infective bites/person per year) [18–20]. In addition, healthy adults living in Kintampo were sampled as representing a semi-immune population.

Informed written consent was obtained from adults and parents or guardians of children. In addition, informed written assent was obtained from children between 12 and 17 years of age. The study protocol received ethical approval after review by the Institutional Review Board of the Noguchi Memorial Institute for Medical Research, University of Ghana and the Ethics Committee of the London School of Hygiene and Tropical Medicine. Venous blood from (1) children with malaria confirmed by rapid diagnostic test and microscopy and (2) healthy adults with no recent history of malaria was collected into tubes containing acid citrate dextrose, from which plasma was obtained.

### Recombinant Antigens

A panel of 9 recombinant antigens based on sequences of key EBA and Rh merozoite invasion ligands was used for assays to study antibodies in plasma samples. Five of the antigens—regions III–V of EBA140 (3D7; amino acids 770–1064), regions III–IV of EBA175 (3D7; amino acids 761–1298), a portion of the EBA181 sequence (3D7; amino acids 769–1365), Rh2.2030 (3D7; amino acids 2027 to 2533, common region for both Rh2a and Rh2b), and Rh4.2 (amino acids 1277–1451)—were expressed in *Escherichia coli* cells and purified using glutathione-agarose beads [21–23]. Four other antigens—full-length ectodomains of EBA140, EBA175, EBA181, and Rh5—were expressed as biotinylated proteins in HEK293E cells and purified by nickel affinity chromatography [24].

### Enzyme-Linked Immunosorbent Assays

Enzyme-linked immunosorbent assays (ELISAs) were performed to determine levels of antigen-specific antibodies in plasma samples. Antibody reactivity to the GST-tagged antigens (EBA140 RIII–V, EBA175 RIII–V, EBA181 RIII–V, Rh2.2030, and Rh4.2) were measured as previously described for other GST-tagged antigens [25], whereas ELISAs using biotinylated antigens (full-length EBA140, EBA175, EBA181, and Rh5) were performed using methods previously described [26], with minor modifications.

In brief, 96-well plates (Immulon 4HBX; Thermo Scientific) were coated at 0.50 µg/mL of individual recombinant antigens (GST-tagged antigens) in coating buffer (15 mm Na_2_CO_3_, 35 mm NaHCO_3_, pH 9.3) and incubated overnight at 4°C. The plates were washed with phosphate-buffered saline (PBS) containing 0.05% v/v Tween 20 (PBS-T) and blocked for 3 hours with 1% skimmed milk (Marvel UK, Thame, UK) in PBS-T at room temperature. For biotinylated antigens, streptavidin-coated 96-well ELISA plates (Nunc, Roskilde, Denmark) were washed with PBS-T and blocked for 30 minutes with PBS containing 0.5% bovine serum albumin at room temperature, before coating with individual recombinant antigens at 0.50 µg/mL. Antigen-coated plates were incubated at room temperature for 45 minutes. Plates were washed before addition of plasma samples diluted 1:500 (GST-tagged antigens) and 1:1000 (biotinylated antigens) dilution in blocking buffer and incubated overnight at 4°C.

The plates were washed, incubated with horseradish peroxidase-conjugated goat antihuman IgG (diluted 1/15 000) (Dako Ltd., Ely, UK), and washed again, before the addition of tetramethylbenzidine substrate solution. After incubation for 20 minutes, reactions were stopped using 0.2 M sulphuric acid, and optical densities (ODs) were measured at 450 nm in a microplate reader (Bio-Rad iMark, Hercules, CA). All plasma samples were assayed in duplicates, and any background OD in wells coated with GST only was subtracted from the antigen-specific OD measurements.

Antibody reactivities of 20 nonimmune individuals (malaria nonexposed Europeans) were tested as negative controls in all assays. Antibody reactivity of a test sample was considered positive if the OD was greater than the mean plus 3 standard deviations of these controls.

### Purification of Total Immunoglobulin G

Total IgG was purified from 50 plasma samples using a protein G affinity column (GE Healthcare, Uppsala, Sweden), according to the manufacturer’s instructions. In brief, the column was equilibrated with 20 mM sodium phosphate buffer (pH 7.0), after which the diluted plasma samples were injected on the column. The column was washed with the equilibration buffer before eluting with 0.1 M glycine buffer (pH 2.8). Concentration of IgG fractions was determined using the Pierce Bicinchoninic acid Protein Assay Kit (Thermo Scientific, Rockford, IL).

### Invasion Assays

Erythrocyte invasion assays were set up using schizont stage parasites according to methods described previously [27], with slight modifications. Parasite cultures with parasitemia between 1% and 5% of predominantly schizont stage parasites (>90%) were used for invasion assays. These were set up by adding the parasite culture to carboxyfluorescein diacetate succinimidyl ester-stained erythrocytes at 2% hematocrit and purified IgG at 5 mg/mL in a 96-well titer plate in duplicates. The plate was incubated at 37°C overnight in a gas mixture of 2% O_2_, 5.5% CO_2_, and 92.5% N_2_. Hoechst 33342 was used to stain parasite deoxyribonucleic acid to differentiate parasitized erythrocytes from uninfected erythrocytes, so that parasite invasion could be determined using flow cytometry on a BD Fortessa X-20.

### Statistical Analyses

Statistical analyses were performed with Minitab (version 17). One-way analysis of variance or Kruskal-Wallis tests were applied for across-group comparisons for normally and nonnormally distributed data, respectively. Post hoc tests of Student *t* test or Mann-Whitney *U* tests were applied for pairwise analysis where necessary. Pearson and Spearman’s rank correlation coefficients were used to assess the association between 2 continuous variables. Multivariate regression analyses were carried out to determine which antigens predicted inhibitory activity. For all analyses, *P* < .05 was considered statistically significant.

## RESULTS

### Demographic Data and Parasitemia Levels of Children With Malaria

Plasma samples were obtained from 423 children with malaria in 3 different areas of Ghana. Children with malaria recruited in Accra (N = 125) were significantly older than those recruited in Navrongo (N = 131) (*P* = .002) (Table 1). Ages of children recruited in Kintampo (N = 167) were not significantly different from those in Accra and Navrongo (*P* = .10 and *P* = .09, respectively). There was no statistically significant difference in proportions of males and females sampled across sites (*P* = .42) (Table 1). Parasite densities differed significantly across sites (*P* < .001) (Table 1), with children from Kintampo showing the highest parasitemias, followed by Navrongo and then Accra, correlating with levels of malaria endemicity in these areas.

**Table 1.** Clinical and Demographic Characteristics of Malaria-Positive Ghanaian Children Aged 2–14 Years in Kintampo, Navrongo, and Accra at the Time of Sampling^a^

Characteristics	Kintampo	Navrongo	Accra	Total	*P* Value
Number of subjects	167	131	125	423	.419^b^
Female (%)	46.70	52.10	43.50	47.50	
Age in years, mean (SE)	5.7 (0.3)	5.1 (0.3)	6.4 (0.3)	5.7 (0.2)	.009^c^
Parasitemia per µL, mean (SE)	137 824 (17 297)	46 863 (4533)	41 184 (3876)	82 189 (7601)	<.001^c^

Abbreviations: ANOVA, analysis of variance; SE, standard errors of the mean.

^a^Relative malaria transmission intensities as measured by entomological inoculation rates in the areas are in the order Kintampo > Navrongo > Accra. Data for age and parasitemia are presented as means (with SE).

^b^ *P* value obtained from χ^2^ test.

^c^ *P* value obtained from ANOVA.

### Antibody Responses of Children With Malaria to Invasion Antigens

**Figure 1.**
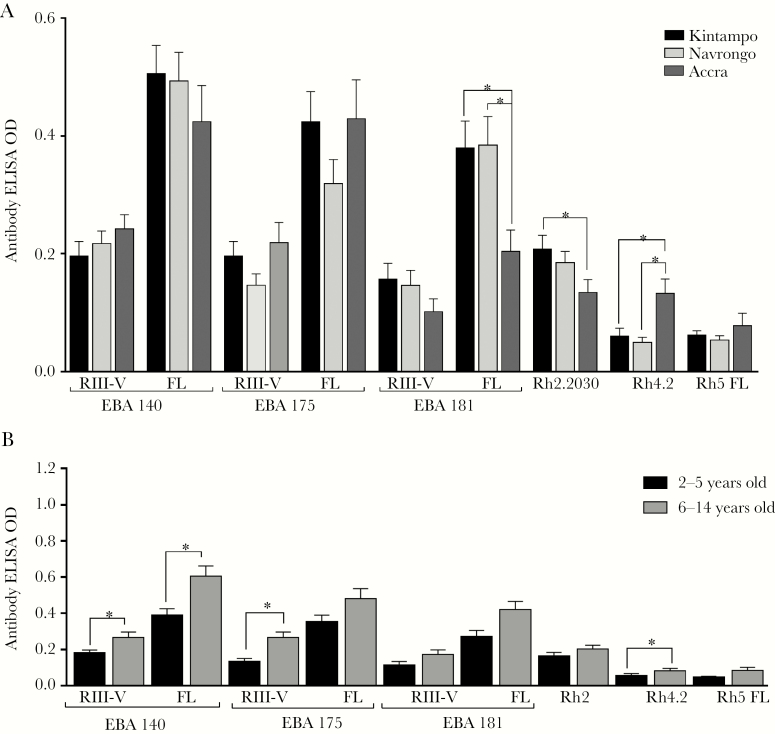
(A) Invasion antibody profiles of children with malaria across 3 endemic regions of Ghana. Antibody reactivity of malaria-positive children to erythrocyte-binding antigen (EBA) and reticulocyte binding-like homolog proteins (Rh) between Kintampo (*N* = 167), Navrongo (*N* = 131), and Accra (*N* = 125). *, Group means differ significantly (*P* < .05, Student’s *t* test). (B) Age-dependent acquisition of immunoglobulin (Ig)G against recombinant EBA and Rh proteins. The IgG reactivity of malaria-positive children (*N* = 423) to the antigens were grouped to 2 age groups: 2–5 years and 6–14 years. Bars represent group means, and error bars represent standard errors of the means. *, Group means differ significantly (*P* < .05, Student’s *t* test). ELISA, enzyme-linked immunosorbent assay; FL, full length; OD, optical density.

To investigate the relationship between antimalarial antibodies and age, parasitemia, and levels of malaria endemicity, levels of antibodies to a panel of *P falciparum* invasion-related antigens were measured in plasma samples from malaria-positive children in Ghana. Antibody ELISA ODs against all antigens tested were compared across the 3 sites. Significant differences among the areas were observed for antibody levels (1) to full-length EBA181 and (2) to the partial-length recombinant proteins Rh2.2030 and Rh4.2. Antibody reactivity to full-length EBA181 was higher in children from Kintampo (*P* = .003) (Figure 1A) and Navrongo (*P* = .003) (Figure 1A) compared with children from Accra. Likewise, antibody reactivity to Rh2.2030 was significantly higher in children living in Kintampo compared with children living in Accra (*P* = .024) (Figure 1A), although no significant difference was observed in comparison to children from Navrongo (*P* > .05) (Figure 1A). In contrast, children in Accra showed higher antibody levels to Rh4.2 compared with children living in Navrongo and Kintampo (*P* < .05 for each comparison) (Figure 1A). There were no statistically significant differences across sites for the remaining antigens.

In endemic areas, age is often a surrogate marker for exposure, so we compared malaria-positive children of 2 age categories: ages 2–5 and 6–14 years old. The older age group had higher levels of IgG for all antigens, and these differences were statistically significant for RIII–V of EBA140 and EBA175, and to full ectodomains of EBA175 and EBA181, as well as to Rh4.2 (*P* < .05 for all comparisons) (Figure 1B). Age-adjusted multivariate analysis was performed to determine the relationship between antibody reactivity, parasite density and level of exposure, using transmission intensity as a marker of exposure. Antibody levels of EBA140 RIII-V, EBA181 RIII-V and PfRh5 were significantly influenced by level of exposure only (*P* < .001 for all analyses; Table 2). Antibody levels to EBA181 FL were influenced by both age (*P* = .003; Table 2) and level of exposure (*P* < .001; Table 2). On the other hand, antibody levels to Rh2.2030 and Rh4.2 were significantly influenced by parasite density (*P* = .002 for both antigens; Table 2) and level of exposure (*P* = .026 and *P* = .037 respectively; Table 2).

**Table 2.** Multiple Linear Regression Analysis of Factors Associated With Anti-Merozoite Antibody Levels in Malaria-Positive Children

	Age		Parasite Density		Level of exposure	
Antigens	F	*P*-value	F	*P*-value	F	*P*-value
**EBA140 RIII-V**	1.62	.203	0.08	.774	**6.96**	**.001** ^**a**^
**EBA140 FL**	1.55	.214	2.36	.125	1.09	.336
**EBA175 RIII-V**	0.14	.704	2.22	.137	1.59	.205
**EBA175 FL**	3.05	.082	**9.01**	**.003** ^**a**^	1.22	.297
**EBA181 RIII-V**	1.7	.193	1.29	.257	**10.26**	**<.001** ^**a**^
**EBA 181 FL**	**9.26**	**.003** ^**a**^	1.44	.231	**12.89**	**<.001** ^**a**^
**Rh2**	0.14	.705	**10.22**	**.002** ^**a**^	**3.68**	**.026**
**Rh4**	0.21	.651	**6.17**	**.002** ^**a**^	**4.39**	**.037**
**Rh5 FL**	1.25	.265	0.06	.807	**12.77**	**<.001** ^**a**^

Table summarizing the multiple linear regression analyses. Antibody levels were the outcome variables, while age, parasitaemia, and transmission intensity (level of exposure) were the predictor variables. F = F-statistic. Statistically significant predictors are shown in bold face. ^**a**^Significant after Bonferroni’s *P* value adjustment. FL=full-length.

### Correlations Between Breadth of Antibody Reactivity, Age, Parasite Density, and Exposure Among Children

**Figure 2.**
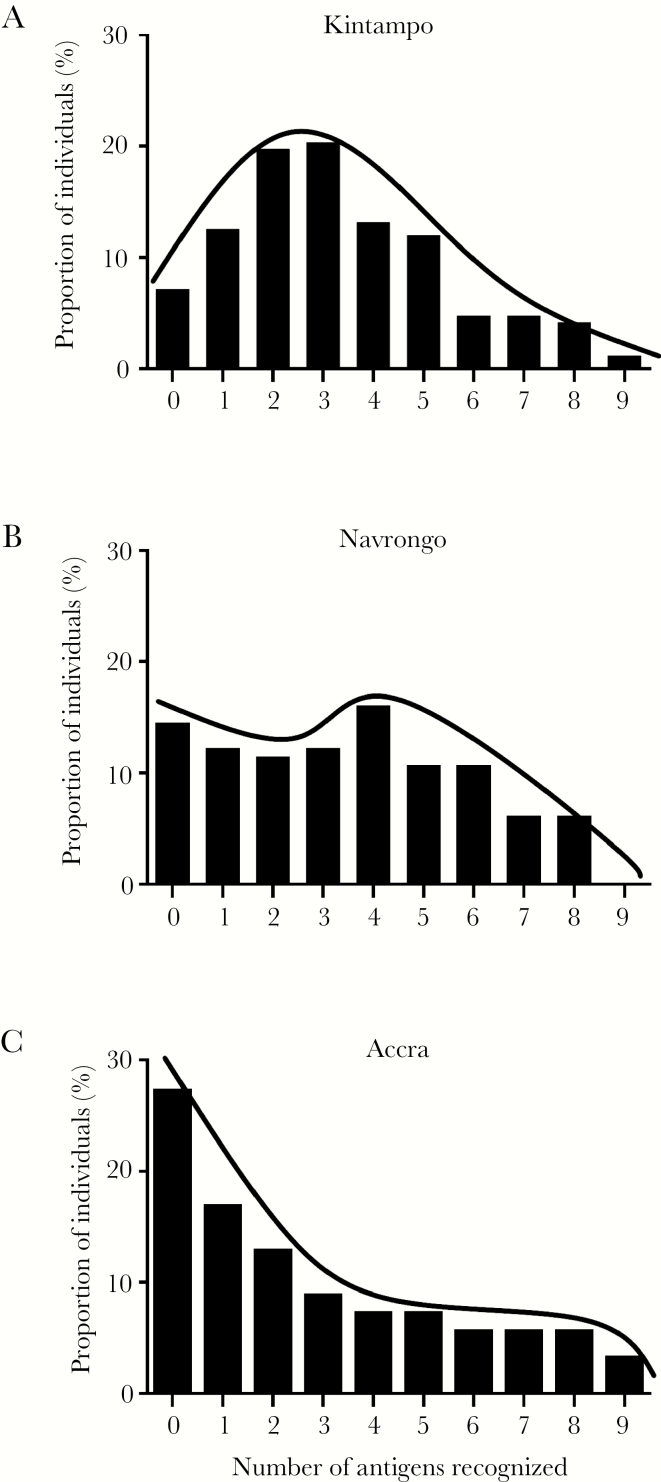
The breadth of antibody reactivity to multiple antigens varies across areas with varying transmission intensity. Breadth scores were calculated for each child based on the number of antibody responses. Distribution of the breadth of antibody reactivity of malaria-positive children was compared between Kintampo (*N* = 167), Navrongo (*N* = 131), and Accra (*N* = 125).

Previous studies have suggested that breadth of antibody reactivity is a correlate of protection and an important predictor of disease outcome, although combinations of specific antibody responses are more strongly associated with protection [9, 26, 28]. Breadth was estimated as the number of recombinant antigens recognized (IgG reactivity above the determined ELISA cutoff level) for each child. The distribution of breadth of antibody reactivity was compared across the 3 endemic areas. The distribution in Kintampo was peaked, approaching a normal distribution with most children recognizing 2–4 antigens (skewness = 0.60, kurtosis = −0.16) (Figure 2A). The distribution in Navrongo showed almost no peak, with an even spread of antigens recognized (skewness = 0.19, kurtosis = −0.99) (Figure 2B). In contrast, for samples from Accra, the distribution showed a left skewed peak (skewness = 0.75, kurtosis = −0.65) (Figure 2C), with most children recognizing less than 3 antigens. In addition, breadth of antibody reactivity was positively correlated with age (ρ = 0.201, *P* < .001). Regression analysis showed that the strongest predictors of breadth of antibody reactivity were age (*F* = 24.01, *P* < .001) and endemic area of sampling (*F* = 4.80, *P* = .009).

Percentage seropositivity of children (IgG reactivity above the cutoff levels) for each of the 9 antigens tested was compared for the malaria cases recruited across the 3 endemic sites. We observed no significant difference in the prevalence of antibodies to RIII–V of EBA140, EBA175, EBA181, and full-length Rh5 (*P* > .05 for all comparisons) (Supplementary Figure 1). However, the proportion seropositive for full-length EBA140, EBA175, EBA181, Rh2.2030, and Rh4.2 differed significantly between the 3 sites, with Accra showing the lowest proportions for all antigens (*P* < .05 for all comparisons) (Supplementary Figure 1).

### Antibody Reactivity to Merozoite Antigens in Adult Donors

**Figure 3.**
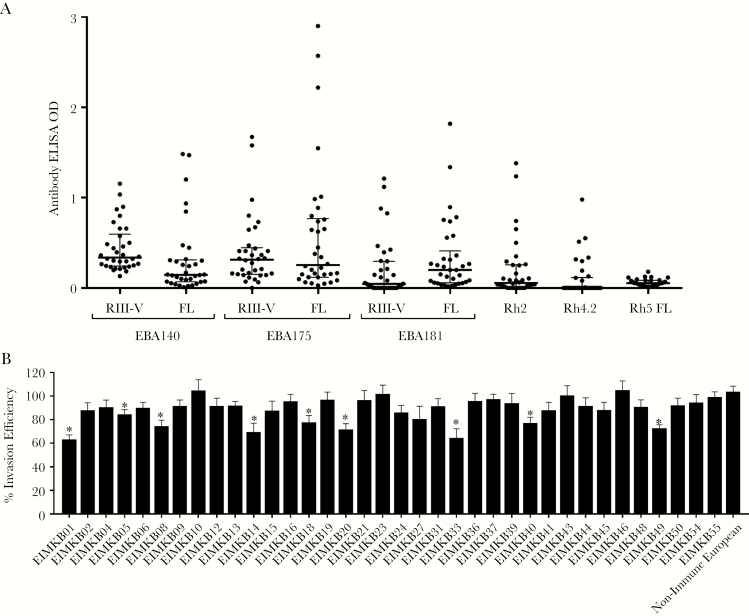
(A) Antibody enzyme-linked immunosorbent assay (ELISA) optical densities (ODs) for serum immunoglobulin (Ig)G against erythrocyte-binding antigen (EBA) and reticulocyte binding-like homolog protein (Rh) antigens in plasma from Ghanaian adult donors. The ELISA assays were conducted using plasma collected from 50 semi-immune adults residing in Kintampo. Data are presented as dot plots. Lines represent medians. Error bars represent interquartile ranges. (B) Average growth inhibitory activity of purified IgG from adult donors against 7 *Plasmodium falciparum* isolates. Growth inhibition assays were set up in duplicate in 96-well plates to test the inhibitory activity of 36 purified IgG preparations (at 5 mg/mL) from semi-immune adults living in Kintampo. Four laboratory strains (3D7, W2Mef, K1, and GB4) and 3 clinical isolates (EIMK084, EIMK239, and EIMK244) were tested. Parasitemia was determined by flow cytometry using BD FACS Fortessa. Data are presented as a percentage of invasion efficiency in comparison to the uninhibited control. Error bars represent standard errors of the means. *, Invasion inhibition by purified IgG is statistically significant relative to uninhibited control (*P* < .05, Student’s *t* test).

Plasma IgG reactivity of the adult donors to the same 9 recombinant antigens was also determined (Figure 3A). All 50 donors were seropositive against 1 or more of the antigens: EBA140 RIII–V (96%), full-length EBA140 (42%), EBA175 RIII–V (78%), full-length EBA175 (60%), EBA181 RIII–V (42%), full-length EBA181 (62%), Rh2 (42%), Rh4 (28%), and Rh5 (26%). Antibody OD levels to full-length and RIII–V fragment antigens were strongly correlated with one another for EBA175 (R = 0.779, *P* < .001) and EBA140 (R = 0.641, *P* < .001), whereas antibody levels to full-length and fragment antigens of EBA181 showed weaker correlation (R = 0.354, *P* = .012). No association was found between donor age and ELISA ODs for any of the antigens tested.

### Invasion Inhibitory Activity of Purified Immunoglobulin G Fractions From Adults

Given the relationship between parasite density and antibody levels observed in children, we sought to further examine the relationship between antibody reactivity to EBA and Rh antigens and functional activity. In vitro invasion inhibitory assays were performed using purified IgG from adults against 4 laboratory-adapted strains (3D7, W2mef, K1, and GB4) and 3 local clinical isolates from Kintampo (EIMK084, EIMK239, and EIMK244). There was sufficient purified IgG amount from 36 of the donors for performing assays on all 7 parasite lines. Across the different lines, purified IgG from individual donors showed an average invasion inhibitory ability of 15%, ranging between 10% and 40% for individual IgG samples (Figure 3B). There were differences in the levels of inhibition measured for the assays performed on different parasite lines (Supplementary Figure 2). For example, the laboratory isolate 3D7 was significantly inhibited by IgG from donors EIMKB33 and EIMKB20 compared with the control (Supplementary Figure 2A and B), whereas W2mef was inhibited by IgG from most individuals. Immunoglobulin G had generally less inhibitory effects in the assays performed on parasite isolates GB4 and K1 (Supplementary Figure 2C and D). Comparing the 3 local parasite isolates, there was more IgG inhibition seen against isolate EIMK239 than isolate EIMK084 and EIMK244 (Supplementary Figure 2E–G). It is unknown whether these differences are due to actual intrinsic variation among the isolates, because each one was not assayed with multiple independent biological preparations. For the measurement of invasion inhibition overall, the mean of the inhibitory activity across all 7 isolates was considered in the subsequent analysis.

### Antibody Reactivity Is Correlated With Invasion Inhibition

**Figure 4.**
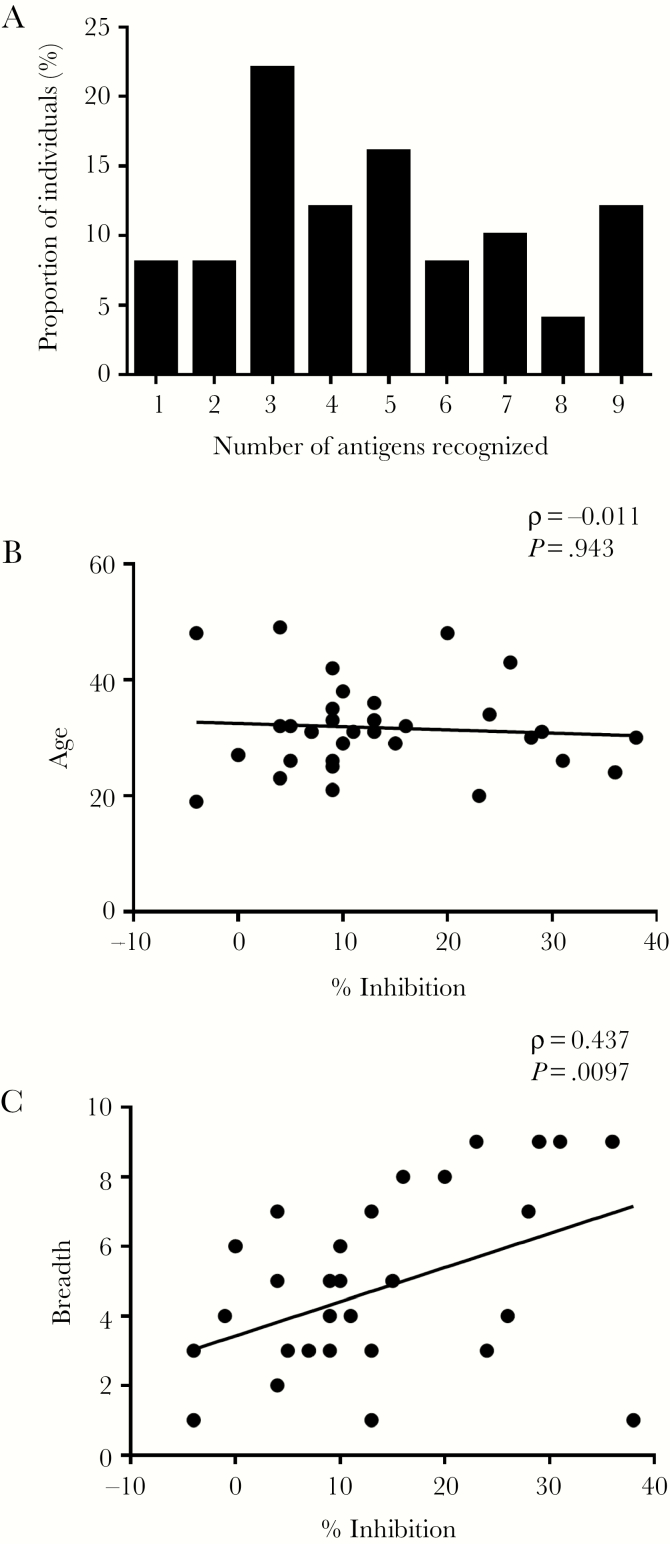
(A) The breadth of antibody reactivity of semi-immune adults from Kintampo against multiple *Plasmodium falciparum* antigens. Breadth scores were calculated for each adult based on the number of antigens recognized by plasma immunoglobulin (Ig)G from individual donors. Individual antigens are as labeled in Figure 1. Bars represent the proportions of individuals recognizing each given number of antigens. (B and C) Breadth of antibody reactivity is associated with in vitro *P falciparum* growth inhibition. Dot plot showing correlation between breadth of antibody reactivity (as shown in Figure 4A) and (B) age and (C) mean inhibition of purified IgG when tested against 7 parasite lines in culture (as shown in Supplementary Figure S2). Correlations of *P* < .05 were considered statistically significant by Spearman rank test.

**Figure 5.**
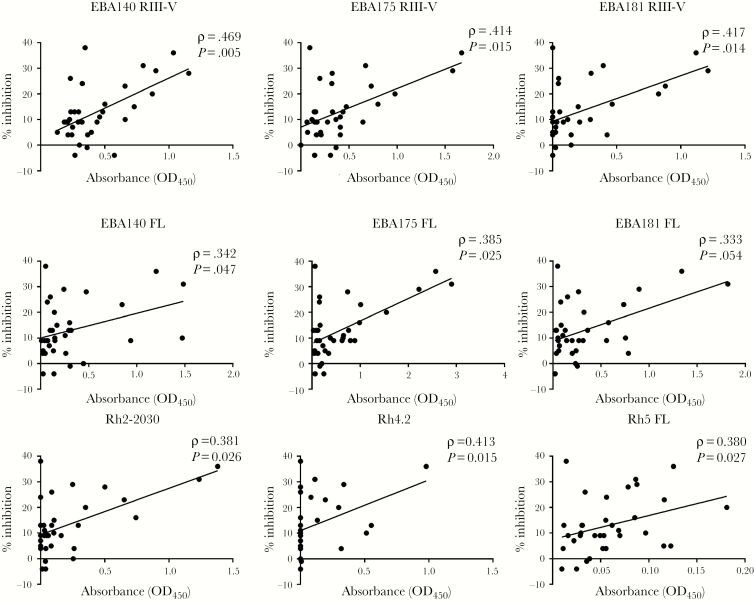
Correlation between reactivity to erythrocyte-binding antigen (EBA) and reticulocyte binding-like homolog protein (Rh) antigens and mean inhibition by purified immunoglobulin (Ig)G. Average invasion inhibition of 7 parasite lines by donor IgG was correlated with reactivity to EBA and Rh antigens. Correlations with *P* < .05 were considered statistically significant by Spearman rank test.

Next, we examined the relationship between antibody reactivity and invasion inhibitory activity in cultured parasites. Breadth of antibody reactivity was calculated based on the number of antigens to which the individual was seropositive (Figure 4A). Although the breadth of antibody response was not correlated with age in adults (Spearman’s rho = −0.011, *P* = .94) (Figure 4B), a significant association was observed between breadth of antibody reactivity and invasion inhibitory activity (Spearman’s rho = 0.437, *P* = .010) (Figure 4C). Furthermore, antibody ELISA OD levels against each of the individual antigens were significantly correlated with invasion inhibition (*P* < .05 for each analysis) (Figure 5).

To explore which specific antigen combinations were associated with the observed invasion inhibitory activity, we grouped the antigens into 2-, 3-, 4-, and 5-way combinations and obtained a breadth score for each combination for each donor, based on seropositivity. The breadth scores for each combination were then correlated with mean invasion inhibitory activity. Several combinations were significantly correlated with invasion inhibition. In general, combinations that included Rh proteins showed strong association with invasion inhibitory activity. After Bonferroni correction, a 3-way combination of Rh2, Rh4, and Rh5 was the most strongly correlated with invasion inhibition (R = 0.55; uncorrected *P* = .00068, corrected *P* < .05) (Supplementary Table 1).

## DISCUSSION

It has previously been shown that antibodies against *P falciparum* merozoite EBA and Rh proteins have greater inhibitory effect against parasites in culture when used in combination [10], supporting the idea that antibodies against multiple antigens can act in combination to provide protection against malaria. Effective naturally acquired immunity may require the development of substantial antibodies to multiple EBA and PfRh antigens. The present study was designed to examine the patterns and repertoire of responses to merozoite invasion antigens in relation to age, exposure, and parasite density in children with malaria. In addition, the relationship between the ability of purified IgG to inhibit parasite growth in vitro and the antibody reactivities to EBA and Rh antigens in semi-immune adults living in an endemic area of Ghana was examined.

Antibody levels to different antigens were weakly or moderately correlated with one another, as previously observed elsewhere [9, 26]. Consistent with previous studies, proportion of seropositive individuals against the Rh proteins, especially Rh4 and Rh5, were generally lower than EBA proteins [6, 9]. Antibody levels also varied significantly across transmission areas, with some increasing with transmission intensity, whereas others decreased. It has been proposed that the differences observed in the rates of acquisition of antibodies to merozoite antigens may be due to differences in the immunogenicity as well as the subcellular localization of the antigens [29]. It is known that antibodies acquired during a malaria transmission season can be lost at different rates over the course a subsequent dry season when there is little to no malaria transmission [30–32]. It has been suggested that the rates of acquisition of antibodies are antigen-specific, and different antigens may elicit antibody-secreting cells with different longevity [29, 33].

Breadth of antibody reactivity to multiple antigens is associated with reduced risk of clinical malaria and is a correlate of protective immunity [34], and combined antibodies to EBA and Rh antigens have been strongly associated with protective immunity [9]. A previous study showed that breadth of antibody response increased with age, suggesting that acquisition of humoral immunity requires repeated exposure to malaria infection [35]. Similar to their finding, breadth of reactivity correlated with age in children with malaria in our study, and that the area of lowest endemicity (Accra) had the lowest breadth of reactivity per individual.

Growth inhibitory activity of purified IgG from a panel of adult donors living in a malaria-endemic area was determined against 7 parasite lines. Consistent with what has previously been reported with IgG from immune donors [36], varying levels of inhibitory activity were observed across donors. A previous study in Kenya reported that plasma levels of antibodies to merozoite surface protein (MSP)3 correlated with growth inhibitory activity against 3 laboratory strains [37]. In the present study, average invasion inhibitory activity against 7 parasite lines was significantly positively correlated with antibody reactivity for each of the EBA and Rh recombinant antigens tested. Furthermore, a combination of antibodies against Rh2, Rh4, and Rh5 emerged as the strongest predictor of overall with growth inhibitory activity against the parasite strains tested.

Purified IgG from some samples seemed to slightly enhance the growth of some parasite lines in vitro, a phenomenon that has previously been reported in other studies, but the mechanism is currently unknown. Some antibodies to recombinant antigens have been shown to enhance merozoite invasion in vitro [38], and it has also been reported that pooled IgG from West African donors enhanced parasite growth in vitro [14]. Another study showed that the majority of IgG preparations from Kenyan donors enhanced parasite growth by varying degrees [39]. It is difficult to determine what could account for this enhancement of growth, but antibody-mediated enhancement of infection has been reported in other pathogens such as human immunodeficiency virus and dengue fever [40, 41]. Recent studies suggested that complement interactions were important for mediating the inhibitory activity of acquired antibodies for many individuals, and invasion enhancement was not prominent if complement was present [42]. Complement-mediated enhancement of parasite growth has also been reported in *P falciparum* using a mouse monoclonal antibody against MSP1 [43].

The present analysis showed that breadth of antibody reactivity to multiple antigens was associated with inhibitory activity of purified IgG, and the individual specific antibody levels were also correlated with inhibition. Results from a birth cohort study in Kenya indicated that there was growth inhibitory activity by IgG but that this did not increase with increasing breadth of antibody reactivity by ELISA [37]. However, our observations support those of others that suggest that breadth of antibody reactivity against different antigens is a key determinant of protection against malaria [2, 28, 44]. One study [45] suggested that parasites grown in the presence of antibodies against 2 vaccine candidate antigens led to the emergence of clones that showed reduced sensitivity to antibody-mediated parasite inhibition. Although such possible selection has not been studied independently, if this effect is real, it would indicate that a multicomponent vaccine may be desirable to prevent the emergence of vaccine resistance.

## CONCLUSIONS

The data presented in this study contribute further evidence in support of using combinations of 2 or more antigens in a potential blood-stage vaccine. Furthermore, our analysis suggests that 3 antigens are optimal, and combinations of Rh2, Rh4, and Rh5 emerged as a potential effective combination.

## Supplementary Data

Supplementary materials are available at *Open Forum Infectious Diseases* online. Consisting of data provided by the authors to benefit the reader, the posted materials are not copyedited and are the sole responsibility of the authors, so questions or comments should be addressed to the corresponding author.

ofz254_suppl_supplementary_figure_1

ofz254_suppl_supplementary_figure_2

ofz254_suppl_supplementary_figure_legends

ofz254_suppl_supplementary_table
