# Supplementary figures and images for: Antibody Reactivity to Merozoite Antigens in Ghanaian Adults Correlates With Growth Inhibitory Activity Against Plasmodium falciparum in Culture
Source: Open Forum Infect Dis. 2019 May 28;6(7):ofz254. doi: 10.1093/ofid/ofz254 (PMC6611546; doi:10.1093/ofid/ofz254)

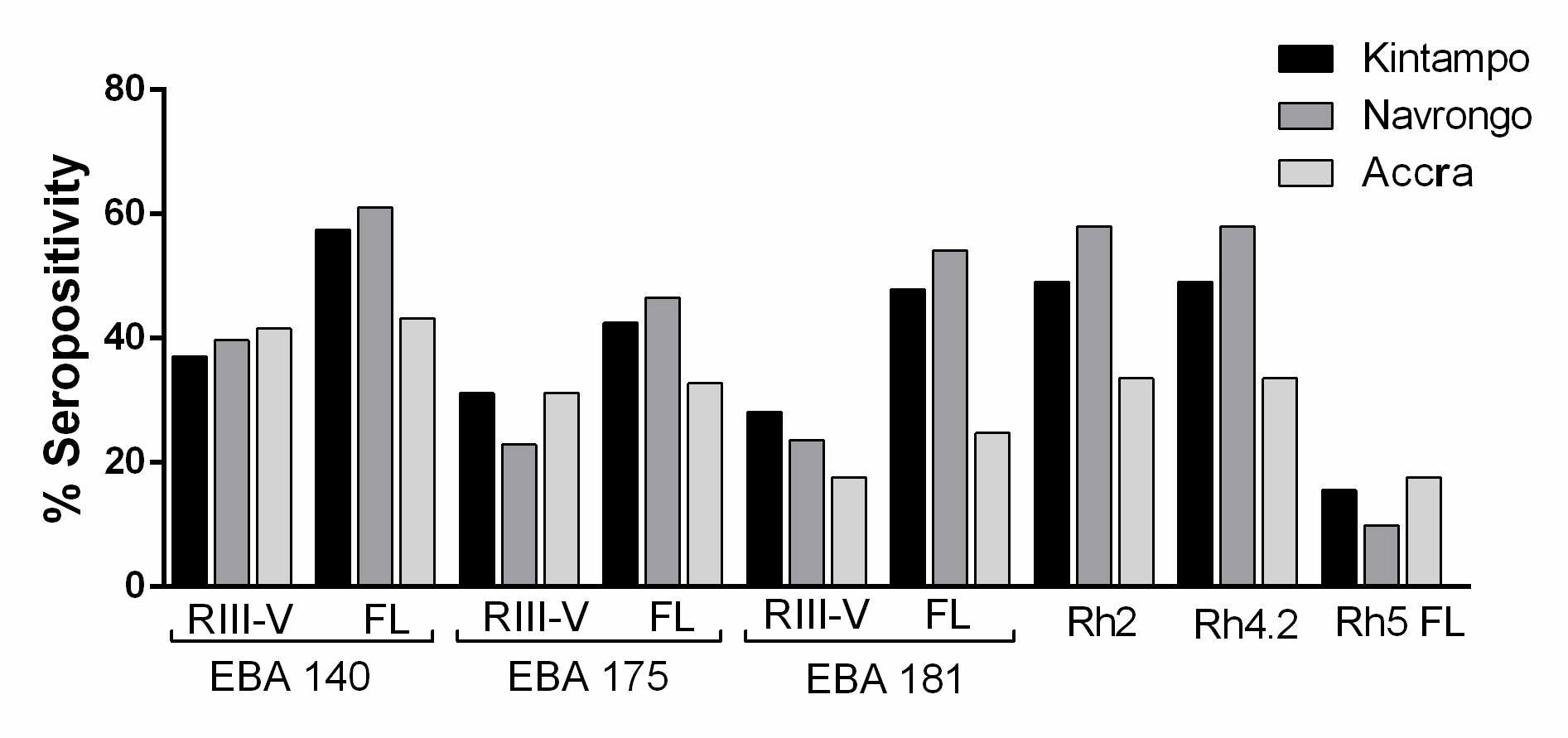

Supplement: ofz254_suppl_supplementary_figure_1 [file ofz254_suppl_supplementary_figure_1.png]

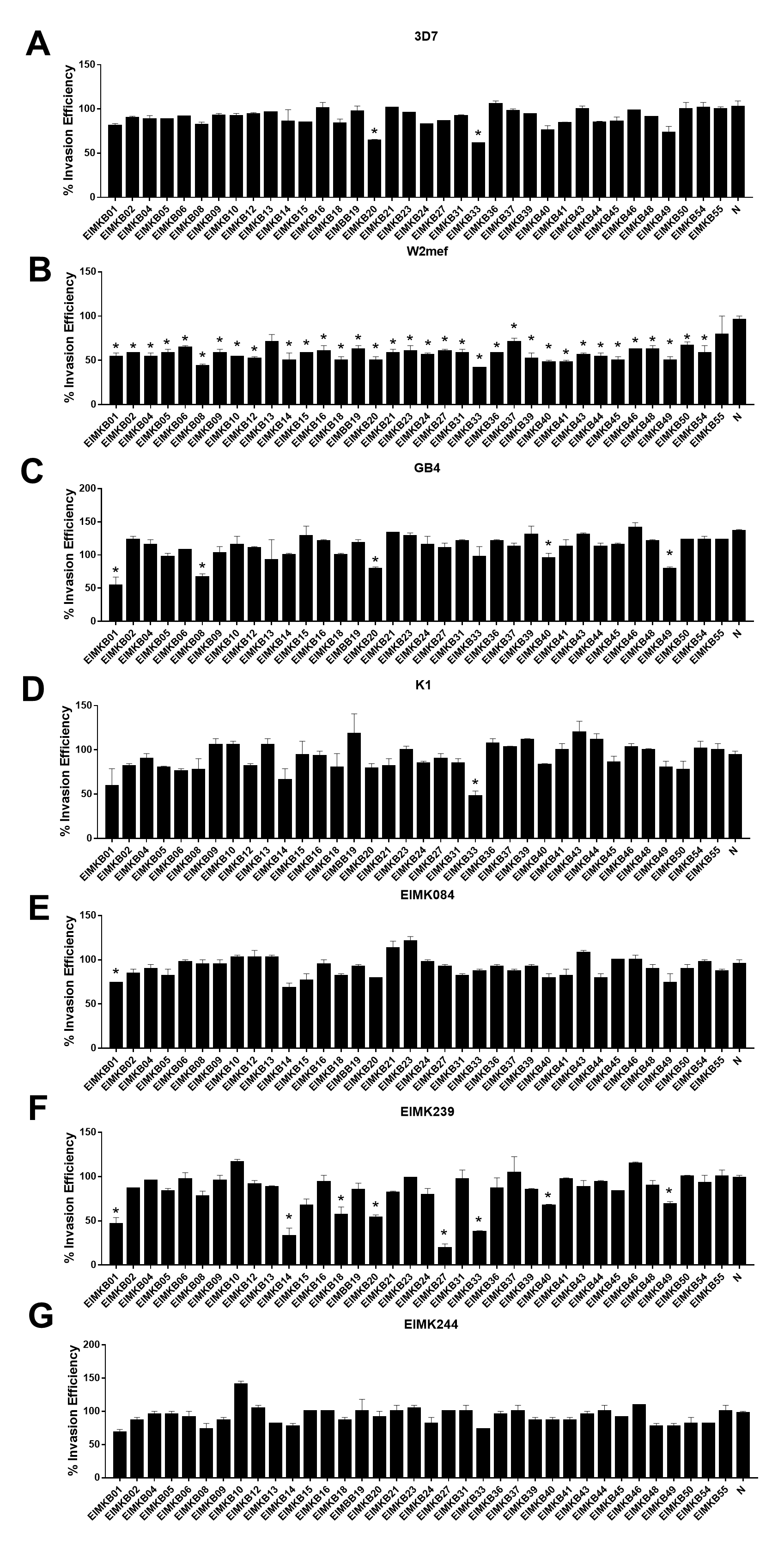

Supplement: ofz254_suppl_supplementary_figure_2 [file ofz254_suppl_supplementary_figure_2.png]
