# Supplementary material for: Antibody Reactivity to Merozoite Antigens in Ghanaian Adults Correlates With Growth Inhibitory Activity Against Plasmodium falciparum in Culture
Source: Open Forum Infect Dis. 2019 May 28;6(7):ofz254. doi: 10.1093/ofid/ofz254 (PMC6611546; doi:10.1093/ofid/ofz254)
Supplement: ofz254_suppl_supplementary_figure_legends [file ofz254_suppl_supplementary_figure_legends.docx]

**Supplementary Figure 1. Seroprevalance of antibodies against EBA and Rh proteins in children with malaria across areas with varying transmission intensity.** Bar chart showing the percentage of individuals from each endemic area with detectable antibodies to EBA and Rh proteins.

**Supplementary Figure 2. Growth inhibitory activity of purified IgG against laboratory strains and clincal isolates.** Growth inhibition assays were set up in duplicate in 96 well plates to test the inhibitory activity of 36 purified IgG preparations (at 5 mg/mL) from semi-immune adults living in Kintampo. Four laboratory strains (A) 3D7, (B) W2Mef, (C) K1 and (D) GB4, and three clinical isolates (E) EIMK084, (F) EIMK239 and (G) EIMK244. were tested. Parasitaemia was determined by flow cytometry using BD FACS Fortessa. Data are presented as a percentage of invasion efficiency in the uninhibited control. Error bars represent standard error of the mean. *Invasion inhibition by purified IgG is statistically significant relative to uninhibited control
