# Supplementary material for: Antibody Reactivity to Merozoite Antigens in Ghanaian Adults Correlates With Growth Inhibitory Activity Against Plasmodium falciparum in Culture
Source: Open Forum Infect Dis. 2019 May 28;6(7):ofz254. doi: 10.1093/ofid/ofz254 (PMC6611546; doi:10.1093/ofid/ofz254)
Supplement: ofz254_suppl_supplementary_table [file ofz254_suppl_supplementary_table.docx]

**SUPPLEMENTARY TABLE 1**

**Supplementary table 1. Correlation between invasion inhibitory activity of IgG and breadth of antibody reactivity for 2, 3 4 and 5 antigen combinations**

|  | **Combination** | **Correlation coeff** | **P-Value** |
| --- | --- | --- | --- |
| Two Antigen Combinations | Rh2 + Rh4 | 0.543 | 0.0009 |
|  | Rh2+EBA140 FL | 0.404 | 0.0180 |
|  | Rh2+EBA175 FL | 0.348 | 0.0440 |
|  | Rh2 +EBA181 FL | 0.358 | 0.0380 |
|  | Rh2+Rh5 | 0.509 | 0.0021 |
|  | Rh4+EBA140 FL | 0.419 | 0.0140 |
|  | Rh4 +EBA175 FL | 0.325 | 0.0610 |
|  | Rh4+EBA181 FL | 0.408 | 0.0170 |
|  | Rh4 + Rh5 | 0.474 | 0.0046 |
|  | EBA140 FL + EBA175 FL | 0.179 | 0.3100 |
|  | EBA140 FL+ EBA181 FL | 0.222 | 0.2100 |
|  | EBA140 FL +Rh5 | 0.379 | 0.0270 |
|  | EBA175 FL + EBA181 FL | 0.155 | 0.3800 |
|  | EBA175 FL + Rh5 | 0.265 | 0.1300 |
|  | EBA181 FL + Rh5 | 0.340 | 0.0490 |
| Three antigen combination | Rh2 + Rh4 + EBA140 FL | 0.501 | 0.0025 |
|  | Rh2 + Rh4 + EBA175 FL | 0.455 | 0.0069 |
|  | Rh2 + Rh4 + EBA181 FL | 0.478 | 0.0042 |
|  | Rh2 + Rh4 + Rh5 | **0.554** | **0.0007** |
|  | Rh2 + EBA140 FL + EBA175 FL | 0.338 | 0.0500 |
|  | Rh2 + EBA140 FL + EBA181 FL | 0.349 | 0.0430 |
|  | Rh2 + EBA140 FL + Rh5 | 0.479 | 0.0041 |
|  | Rh2 + EBA175 FL + EBA181 FL | 0.316 | 0.0690 |
|  | Rh2 + EBA175 FL + EBA181 FL | 0.416 | 0.0140 |
|  | Rh2 + EBA181 FL + Rh5 | 0.441 | 0.0091 |
|  | Rh4 + EBA140 FL + EBA175 FL | 0.331 | 0.0560 |
|  | Rh4 + EBA140 FL + EBA181 FL | 0.376 | 0.0280 |
| Three antigen combinations | Rh4 + EBA140 FL + Rh5 | 0.469 | 0.0051 |
|  | Rh4 + EBA175 FL + EBA181 FL | 0.325 | 0.0610 |
|  | Rh4 + EBA175 FL + Rh5 | 0.385 | 0.0240 |
|  | Rh4 + EBA181 FL + Rh5 | 0.456 | 0.0067 |
|  | EBA140 FL + EBA175 FL + EBA181 FL | 0.199 | 0.2600 |
|  | EBA140 FL + EBA175 FL + Rh5 | 0.294 | 0.0920 |
|  | EBA140 FL +EBA181 FL + Rh5 | 0.338 | 0.0500 |
|  | EBA175 FL + EBA181 FL + Rh5 | 0.276 | 0.1100 |
| Four antigen combinations | Rh2 + Rh4 + EBA140 FL + EBA175 FL | 0.429 | 0.0110 |
|  | Rh2 + Rh4 + EBA140 FL + EBA181 FL | 0.446 | 0.0081 |
|  | Rh2 + Rh4 + EBA140 FL + Rh5 | 0.531 | 0.0012 |
|  | Rh2 + Rh4 + EBA175 FL + EBA181 FL | 0.418 | 0.0140 |
|  | Rh2 +Rh4 + EBA175 FL +Rh5 | 0.479 | 0.0041 |
|  | Rh2 + Rh4 + EBA181 FL + Rh5 | 0.511 | 0.0020 |
|  | Rh2 + EBA140 FL + EBA175 FL + EBA181 FL | 0.314 | 0.0710 |
|  | Rh2 + EBA140 FL + EBA175 FL + Rh5 | 0.403 | 0.0180 |
|  | Rh2 + EBA140 FL + EBA181 FL + Rh5 | 0.421 | 0.0130 |
|  | Rh2 + EBA175 FL + EBA181 FL + Rh5 | 0.384 | 0.0250 |
|  | Rh4 + EBA140 FL + EBA175 FL + EBA181 FL | 0.321 | 0.0640 |
|  | Rh4 + EBA140 FL + EBA175 FL+ Rh5 | 0.389 | 0.0230 |
|  | Rh4 + EBA140 FL + EBA181 FL + Rh5 | 0.434 | 0.0100 |
|  | Rh4 + EBA175 FL + EBA181 FL + Rh5 | 0.382 | 0.0260 |
|  | EBA140 FL + EBA175 FL + EBA181 FL + Rh5 | 0.289 | 0.0980 |
| Five antigen combinations | Rh2 + Rh4 + EBA140 FL + EBA175 FL + EBA181 FL | 0.399 | 0.0200 |
|  | Rh2 + Rh4 + EBA140 FL + EBA175 FL + Rh5 | 0.463 | 0.0059 |
|  | Rh2 + Rh4 + EBA140 FL + EBA181 FL + Rh5 | 0.485 | 0.0036 |
|  | Rh2 + Rh4 + EBA175 FL + EBA181 FL + Rh5 | 0.452 | 0.0073 |
|  | Rh2 + EBA140 FL + EBA175 FL + EBA181 FL + Rh5 | 0.374 | 0.0290 |
|  | Rh4 + EBA140 FL + EBA175 FL + EBA181 FL + Rh5 | 0.375 | 0.0290 |

Table summarizing correlation between breadth of reactivity for 2, 3 4 and 5 antigen combinations and invasion inhibitory activity. Combinations that remain significant after Bonferroni’s *P* value adjustment are shown in bold face. FL=full-length.
